# Detection and Localization of Solid Tumors Utilizing the Cancer-Type-Specific Mutational Signatures

**DOI:** 10.3389/fbioe.2022.883791

**Published:** 2022-04-25

**Authors:** Ziyu Wang, Tingting Zhang, Wei Wu, Lingxiang Wu, Jie Li, Bin Huang, Yuan Liang, Yan Li, Pengping Li, Kening Li, Wei Wang, Renhua Guo, Qianghu Wang

**Affiliations:** ^1^ Jiangsu Cancer Hospital, Jiangsu Institute of Cancer Research, The Affiliated Cancer Hospital of Nanjing Medical University, Nanjing, China; ^2^ Department of Bioinformatics, Nanjing Medical University, Nanjing, China; ^3^ Institute for Brain Tumors, Jiangsu Collaborative Innovation Center for Cancer Personalized Medicine, Nanjing Medical University, Nanjing, China; ^4^ Department of Thoracic Surgery, The First Affiliated Hospital of Nanjing Medical University, Nanjing, China; ^5^ Department of Oncology, The First Affiliated Hospital of Nanjing Medical University, Nanjing, China

**Keywords:** cancer biomarkers, cancer diagnosis, cancer localization, mutational signatures, liquid biopsy

## Abstract

Accurate detection and location of tumor lesions are essential for improving the diagnosis and personalized cancer therapy. However, the diagnosis of lesions with fuzzy histology is mainly dependent on experiences and with low accuracy and efficiency. Here, we developed a logistic regression model based on mutational signatures (MS) for each cancer type to trace the tumor origin. We observed MS could distinguish cancer from inflammation and healthy individuals. By collecting extensive datasets of samples from ten tumor types in the training cohort (5,001 samples) and independent testing cohort (2,580 samples), cancer-type-specific MS patterns (CTS-MS) were identified and had a robust performance in distinguishing different types of primary and metastatic solid tumors (AUC:0.76 ∼ 0.93). Moreover, we validated our model in an Asian population and found that the AUC of our model in predicting the tumor origin of the Asian population was higher than 0.7. The metastatic tumor lesions inherited the MS pattern of the primary tumor, suggesting the capability of MS in identifying the tissue-of-origin for metastatic cancers. Furthermore, we distinguished breast cancer and prostate cancer with 90% accuracy by combining somatic mutations and CTS-MS from cfDNA, indicating that the CTS-MS could improve the accuracy of cancer-type prediction by cfDNA. In summary, our study demonstrated that MS was a novel reliable biomarker for diagnosing solid tumors and provided new insights into predicting tissue-of-origin.

## Introduction

An accurate cancer diagnosis is crucial for choosing the optimal therapy and predicting clinical outcomes (Jerjes et al., 2010; Varadhachary and Raber, 2014; Thomson, 2018). Histological examination of the resected specimen remains the gold standard for diagnosing tumors. However, rapid, accurate diagnosis based on morphology and routine ancillary techniques is challenging for lesions with fuzzy histology, especially metastatic cancers (Saudemont et al., 2018; Conway et al., 2019). The accuracies of computed tomography and positron emission tomography in identifying the tissue-of-origin of the carcinoma with unknown primary were 20–27% and 24–40%, respectively, which are far from enough for determining targeted therapies (Fu et al., 2019; He et al., 2020a). Therefore, effective strategies are urgently needed for tumor detection and localization.

The mutation data is easily accessible molecular profile, which could be robustly retrieved and sequenced in various samples, such as formalin-fixed and paraffin-embedded specimens. Previous studies showed a high concordance in mutational patterns between primary and metastatic tumors, especially when pathogenic mutations in driver genes were considered (Manca et al., 2019). Accordingly, some methods were proposed for tumor origin prediction based on somatic mutations (Dietlein and Eschner, 2014; Marquard et al., 2015; Jiao et al., 2020). However, somatic mutations also could be detected in healthy individuals (Welch et al., 2012; Blokzijl et al., 2016; Martincorena and Campbell, 2016), increasing the difficulty of cancer diagnosis. Moreover, mutational profiles showed substantial overlap across different cancer types, making it difficult to trace the origin of the tumor (Jurmeister et al., 2019).

Somatic mutations result from multiple mutational processes, including exposure to exogenous or endogenous mutagens, enzymatic modification of DNA, and defective DNA repair. Different mutational processes generate unique combinations of mutation types, termed mutational signatures (MS). Single nucleotide variants can be divided into six types according to the type of base substitution: C > A, C > G, C > T, T > A, T > C, T > G. Alexandrov et al. extended the original classification of six types of single-base substitutions by including base 5′ and base 3′ to the somatic mutation. Mutational signature (MS) is created by counting the number of substitutions for each of these 96 mutation types. The COSMIC database has described 30 types of reference MS based on the analyses of ∼10,000 whole-genome or whole-exon sequencing datasets from TCGA and ICGC databases (https://cancer.sanger.ac.uk/signatures/signatures_v2/). MS is cancer-derived etiologies that provide a powerful alternative for understanding cancer pathophysiology (Alexandrov et al., 2013; Helleday et al., 2014; Roberts and Gordenin, 2014; Alexandrov et al., 2016; Pilati et al., 2017; Zou et al., 2018). Unlike the extensive heterogeneity of somatic mutations across samples, MS is more stable across individuals in the same tumor type. Previous studies reported that different tumor types leave distinctive patterns of MS (Degasperi et al., 2020). For example, the MS patterns generated in experimental systems for tobacco carcinogens exposure were observed in lung cancer (Alexandrov et al., 2016). MS patterns in colorectal cancer are mostly related to defective DNA mismatch repair (Pandey et al., 2019). Therefore, we reasonably speculated that MS patterns could predict the tumor origin.

Based on the MS patterns, we used the logistic regression method to construct a model for each cancer type to predict the origin. Our results showed that MS could distinguish cancer patients from healthy individuals and inflammation. Furthermore, our MS-based models showed high accuracy in detecting the origin of tumors in both primary and metastatic lesions. Besides, we also found that MS had a better performance in distinguishing various cancer types than somatic mutations. Finally, we indicated that considering the MS patterns could help increase the accuracy of cancer-type prediction by cfDNA.

## Materials and Methods

### Collection of the Whole Exome Data of Tissues and cfDNA

All variant data of primary tumors were downloaded from TCGA (http://gdac.broadinstitute.org/), International cancer genome consortium (ICGC, https://icgc.org/), and other previous studies (Supplementary Tables S1, S2). In these cases, we used only the data in TCGA for training (Data Set1). The data outside of TCGA were validated (Data set 2). The somatic profiles of metastatic tumors were derived from 303 metastatic tumors across nine tumor types (Supplementary Table S3). We assembled several sets of normal or inflammatory tissues to evaluate the difference in genomic landscape between tumor patients and healthy individuals. One of the data sets included 28 healthy individuals, 48 patients with ulcerative colitis, and 18 patients with colitis-associated neoplasia, and the other data set contained 9 normal brains tissues, 13 normal colon tissues, and 13 normal kidney tissues. We also acquired somatic mutations from 27 breast and 14 prostate cancers of cfDNA and biopsy. All these data were obtained by whole-exome sequencing and aligned to the hg19 genome.

### Identification of the Cancer-Type-Specific Mutational Signatures Patterns

The characteristic MS patterns of each cancer type meet the following requirements. First, MS was observed in at least 20% of samples. Secondly, there were significant differences compared with other cancer types, including a fold change greater than 1.5 and an absolute difference greater than 0.1.

### Mutational Signatures-Based Machine Learning Procedure for Predicting the Cancer Types of the Primary Tumor

For each of the ten cancer types selected from the TCGA data set, we used a stepwise logistic regression model to train classifiers for each cancer type on the CTS-MS described in the above section and validated our models in an independent dataset. To evaluate the performance of our model in different populations, we downloaded the somatic mutation data for Asian populations from the ICGC database, including non-small cell lung cancers (*n* = 76), colorectal cancers (*n* = 187), bladder cancers (*n* = 103), gastric cancers (*n* = 10), and liver cancers (*n* = 163). We developed a logistic regression model based on MS for each cancer type to trace the tumor origin. Take breast cancer as an instance, we calculated the score of each sample in the validation dataset using the breast cancer model, labeling breast cancer patients as “1” and non-breast cancer patients as “0” to obtain grouping information. The prediction performance of AUC was calculated using the predicted values estimated by the model with the combination of selected MS as predictors and the group as an outcome.

### Tracing the Origin of Metastatic Sites Based on Mutational Signatures Patterns

First, we used the liver cancer model above to distinguish primary liver tumors and malignant liver lesions originating from other tissues. We further predicted the origin of lesions originating from other tissues, which were correctly classified in the previous step, including 28 breast cancers, 9 esophagus cancers, and 10 prostate cancers. To predict the origin of malignant liver lesions originating from other tissues, we combined CTS-MS and the score of these three primary tumor models to train a classifier by neural networks based on the three cancer types selected from the TCGA data set. Then, we used the model to predict the origin of malignant liver lesions originating from other tissues.

### Combination of Mutational Signatures Patterns and Somatic Mutation to Distinguish Different Cancer Types Based on Plasma cfDNA Data

Based on the CTS-MS, we predicted the origin of tumors from cfDNA, including 27 breast cancers and 14 prostate cancers. We compared the scores of each sample in the breast cancer model and the prostate cancer model. The origin of the sample was considered to be from the tumor type with a high score. Further, we combined CTS-MS and tumor-specific mutations to improve the precision. We identified the tumor-specific mutations as follows: 1) we calculated the frequency of mutations in each gene in each cancer and identified genes that were mutated in more than 5% of the samples as candidate markers. 2) it was considered a tumor-specific mutation if the mutation frequency changes more than 0.1 compared with other cancer types. Then, using the stepwise logistic regression model, we developed classifiers for prostate and breast cancer based on the CTS-MS and tumor-specific mutations.

### Statistical Analysis

The deconstructSigs approach was used to determine the linear combination of pre-defined signatures of a single tumor sample (Rosenthal et al., 2016). We next applied SomaticSignatrues to identify the *de novo* MS (Gehring et al., 2015). The information of pre-defined MS was downloaded from the COSMIC database. The *de novo* MS was mapped to pre-defined MS through cosine similarity. If the similarity was higher than 0.75, it was considered the same MS.

We annotated the mutated genes in each sample in the STRING database (https://string-db.org/). According to the STRING database, we constructed a network of protein-protein interactions for all mutated genes in each sample. Mutation connection scores were defined by gene connectivity, measured by the ratio of the number of genes with interactions to the total number of mutated genes (Eq. 1). Larger mutation connection scores indicate that the mutated gene is more functionally relevant.

$$\begin{array}{c} Mutation\;connection\;score=\frac{the\;number\;of\;genes\;with\;interactions}{\;the\;total\;number\;of\;mutated\;genes} \end{array}$$ (1)

We calculated the similarity between tumors as Eq. 2. For each sample i of tumor M and each sample j of tumor N, we calculated the cosine similarity (rho) between i and j based on pre-defined MS. Finally, a similarity matrix with m rows and n columns was generated. We performed zero-mean normalization on each row and each column of the similarity matrix. Then, we ranked each row and divided it by the number of columns. Further, we ranked each column and divided it by the number of rows.

$$\begin{array}{c} similarity=\underset{i\leq m}{\sum }\frac{\sum_{j\leq n}rho{\left(i,j\right)}^{2}}{mn} \end{array}$$ (2)

Statistical analyses were performed using R software. The significance probability (p) values were calculated by the two-tailed Wilcoxon test functions in R, and the LSAfun package calculated the cosine similarity. Figures were drawn using the ggplot2, or package under R environment.

## Results

### Mutational Signatures Patterns Distinguish Cancers From Inflammation and Healthy Individuals

**FIGURE 1:**
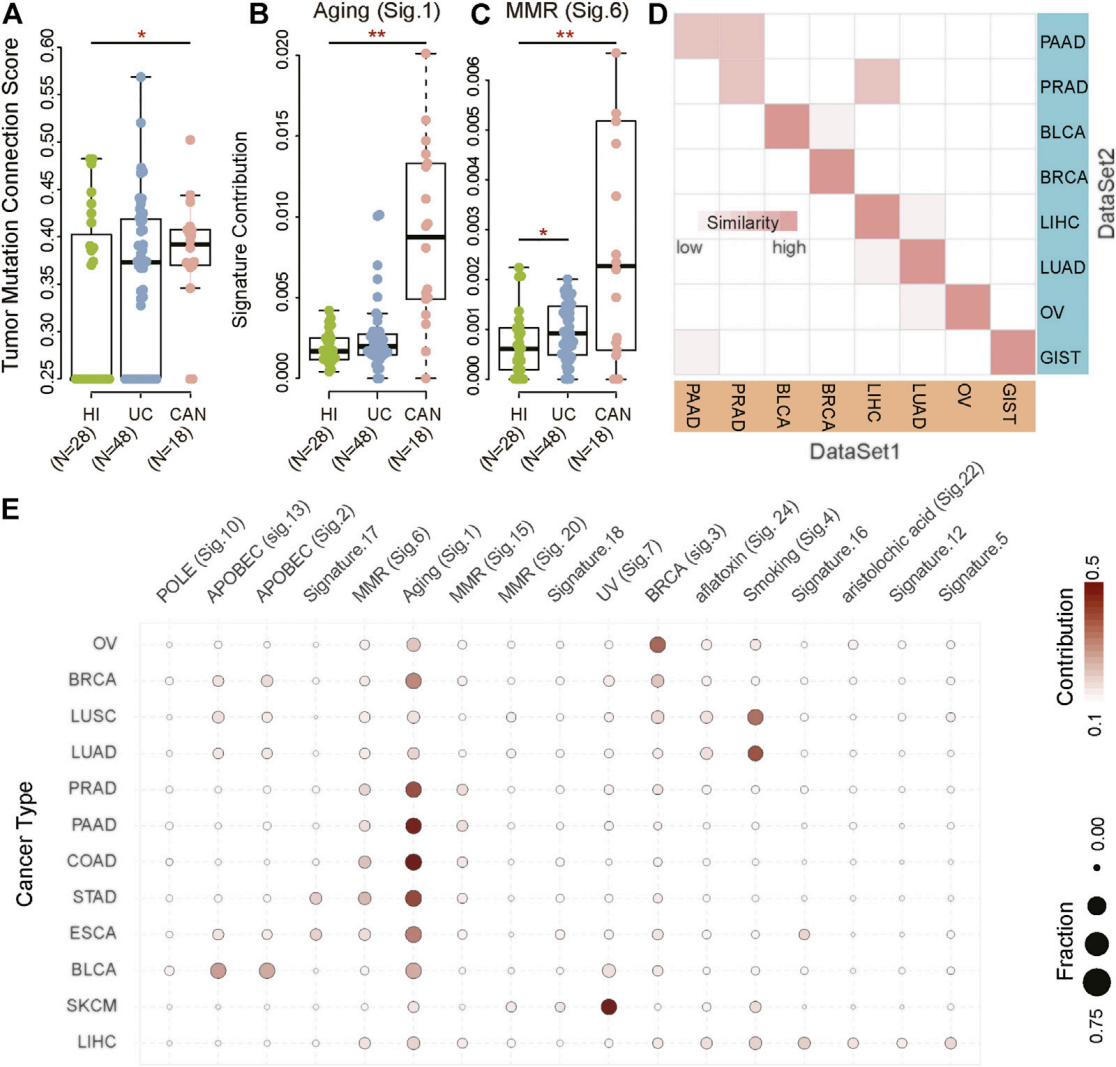
Mutational signatures for cancer diagnosis. **(A–C)** The biological processes of accumulated mutations in healthy individuals (HI) and patients with ulcerative colitis (UC) and colitis-associated neoplasia (CAN). **(D)** The correlation between DataSet1 (TCGA) and DataSet2 (previous studies) based on MS in bladder cancer (BLCA), non-small cell lung cancer (NSCLC), pancreatic cancer (PAAD), breast cancer (BRCA), ovarian serous cystadenocarcinoma (OV), liver hepatocellular carcinoma (LIHC), and gastrointestinal cancer, including colorectal cancer (CRC), esophageal carcinoma (ESCA), and stomach adenocarcinoma (STAD). The darker the color, the higher the similarity. **(E)** Heatmaps of MS in BLCA (*n* = 412), NSCLC (*n* = 1,108), PAAD (*n* = 179), BRCA (*n* = 985), OV (*n* = 435), skin cutaneous melanoma (SKCM, *n* = 468), LIHC (*n* = 464), CRC (*n* = 398), ESCA (*n* = 184), and STAD (*n* = 439). The color indicates the average contribution of MS. The size of the dots indicates the fraction. Fraction: The proportion of samples with a mutational signature contribution of more than 0.06 in each cancer type as a proportion of the total samples. Contribution: Average contribution of each mutational signature in each cancer type.

To compare the difference in the genomic landscape among tumor patients, non-tumor inflammation patients, and healthy individuals, we collected three datasets, including healthy individuals (HI, *n* = 28), patients with ulcerative colitis (UC, *n* = 48), and patients with colitis-associated neoplasia (CAN, *n* = 18) (Nanki et al., 2020). We first computationally defined a tumor mutation connection score measurement to explore whether the mutated genes were functionally related. The higher the tumor mutation connection score, the stronger the functional relevance of the mutated genes in the individual (detail in methods). Results showed that the functional relevance of the mutated genes in CAN is significantly different from HI and UC (Figure 1A). The tumor mutation connection score of CAN was significantly higher than HI and UC (HI vs. CAN, Wilcoxon rank-sum test *p* < 0.001; UC vs. CAN, Wilcoxon rank-sum test *p* < 0.001), indicating that rather than randomly mutation, specific endogenous or exogenous factors were involved in the mutation genesis in CAN. Accordingly, we next explored the potential causal factors of the differences between CAN and HI/UC. Using the non-negative matrix factorization method, we identified two known MS that showed differential contributions among cancer, normal, and inflammation groups (Figures 1B,C; Supplementary Figure S1A), one of which is related to aging and the other is associated with DNA mismatch repair defective (MMR). The contribution of aging-related MS was remarkably higher in CAN than in HI and UC (HI vs. CAN, Wilcoxon rank-sum test *p* < 0.001; UC vs. CAN, Wilcoxon rank-sum test *p* < 0.001, Figure 1B). To avoid bias from age, we checked the distribution of age across three groups in our dataset. There were no differences in the age distribution of the tumor and healthy individuals/inflammation patients (ANOVA test, *p* = 0.319, Supplementary Figure S1B). Furthermore, we found that senior individuals were biased towards higher age-related signature in the healthy population (Spearman correlation:0.41, *p* = 0.035, Supplementary Figures S1C,D). However, there was no correlation between age and the age-related signature of cancer patients (Spearman correlation -0.16, *p* = 0.51, Supplementary Figures S1C,D). Notably, the age-related signature of tumor patients was much higher than those of healthy individuals/inflammatory patients across all age groups. Even the weights of age-related signature in younger tumor patients were five times higher than that in healthy senior individuals (Wilcoxon rank-sum test *p* = 0.004, Supplementary Figure S1C). The MMR-related MS in CAN also showed a higher contribution than HI and UC (HI vs. CAN, Wilcoxon rank-sum test *p* = 0.046; UC vs. CAN, Wilcoxon rank-sum test *p* = 0.007, Figure 1C). These results suggested that the underlying specific mutagenic processes drove the mutations in CAN, which differed from HI and UC. To further validate this observation, we identified MS from 35 normal tissues of the brain (*n* = 9), colon (*n* = 13), kidney (*n* = 13) (Hoang et al., 2016). Results showed that the identified MS had low similarity with any known MS in the Catalogue of somatic mutations in cancer (COSMIC) database (cosine similarity < 0.75). Although somatic mutations were detected in nearly all normal samples, even with some mutations located on cancer driver genes, we did not find any known MS associated with tumor initiation in the whole-exome data of normal tissue (Supplementary Figures S1E,F). These results suggested that most mutations in normal tissues accumulated passively and randomly, without clear evidence of external pathogenic mutagenic processes. Therefore, our results indicated that MS possessed the potential to distinguish cancer patients from inflammation patients and healthy individuals.

### Identification of the Cancer-Type-Specific Mutational Signatures Patterns

We next attempted to evaluate the cancer-type-specificity of MS patterns. We collected two independent datasets with ten primary cancer types, including non-small cell lung cancer (NSCLC), ovarian serous cystadenocarcinoma (OV), bladder cancer (BLCA), breast cancer (BRCA), liver cancers (LIHC), stomach adenocarcinoma (STAD), esophageal carcinoma (ESCA), colon adenocarcinoma (COAD), pancreatic cancer (PAAD), and prostate cancer (PRAD) (Supplementary Table S1). Results showed that tumor samples from the same tissue origins had a high degree of homogeneity in MS between two independent datasets (Figure 1D). In addition to PAAD and PRAD, the MS of other cancer types had been maintained in a stable state (similarity > 0.95). Although the MS of PAAD and PRAD had a slight inconsistency in the two datasets, the similarity of tumors from the same tissue origin was still greater than 0.9. These results suggested that although driver mutations among different individuals were highly diverse, the mutagenic processes in specific cancer types were consistent. Therefore, it was reasonably speculated that MS was a stable and informative tissue-specific molecular biomarker to distinguish cancer types.

To characterize the landscape of MS in cancers, we identified cancer-type-specific MS (CTS-MS) patterns from The Cancer Genome Atlas (TCGA) dataset (DataSet1). The result showed that the contribution of signatures across different cancer types was distinct (Figure 1E; Supplementary Figure S2). Specifically, NSCLC highlighted smoking signature, which was previously found in multiple types of lung cancers with probable etiology of tobacco carcinogens (Pfeifer 2010). OV harbored signature associated with the BRCA1 and BRCA2 mutation (Yang et al., 2018). The most common MS in BLCA was related to the misdirected activity of APOBEC3 cytidine deaminases, especially APOBEC3A or APOBEC3B (Robertson et al., 2018). APOBEC related signature and BRCA-mutation-related signature were the main mechanisms of mutations in BRCA. The risk of skin cancer was associated with UV light exposure (Pham et al., 2020). Signatures related to aflatoxin and aristolochic acid were observed in LIHC (Li et al., 2020; Lu et al., 2020; Zhang et al., 2020). STAD and ESCA were enriched in MMR (Meier et al., 2019; Li et al., 2020). The difference in genomic fingerprints between STAD and ESCA was Signature.16, which currently had no clear exposure factor (Wei et al., 2021). The mutations in COAD resulted from Signature.1, which was associated with an endogenous mutational process initiated by spontaneous deamination of 5-methylcytosine (Pandey et al., 2019). In summary, our results indicated that CTS-MS implied the origin of the tumors and could be possibly used to detect and localize the cancers.

### Mutational Signatures-Based Machine Learning Model for Sensitive Primary Tumor Detection and Classification

**FIGURE 2:**
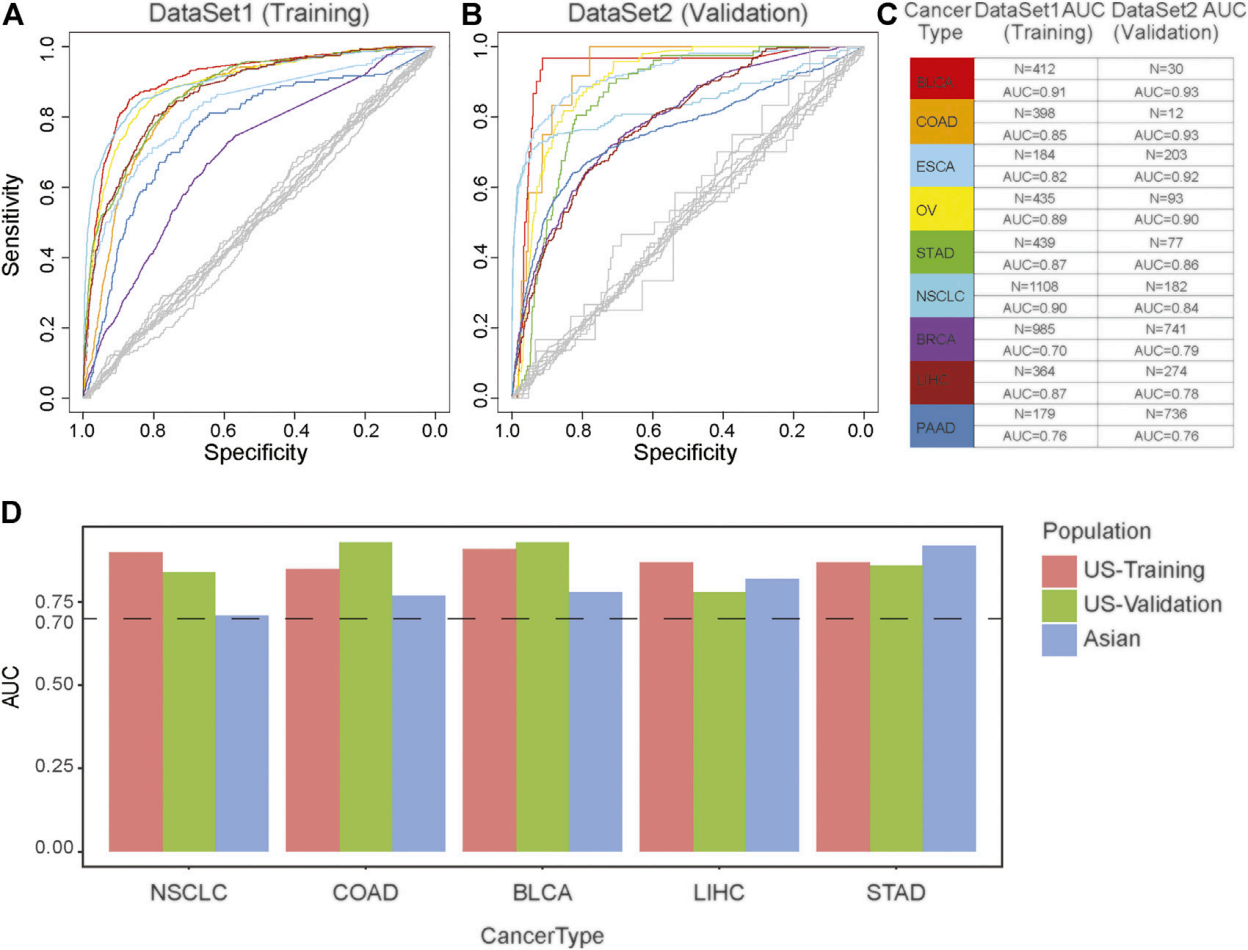
The effectiveness of the cancer diagnosis model based on the MS of the primary tumor. **(A,B)** AUC-curve of cancer diagnosis models in both training **(A)** and validation **(B)** cohort. Random classifiers, indicating the classification accuracies obtained by chance, are shown in gray. **(C)** The value of AUC and the number of patients in both training (left) and validation (right) cohort. **(D)** The model performance across different populations. The vertical axis is the AUC of model. The horizontal axis represents tumor type. Red represents European and American population in the training dataset; Green represents European and American population in the validation dataset; Blue represents Asian populations.

To evaluate the performance of MS in cancer diagnosis, we developed a predictive model for each cancer type based on the TCGA databases, including BLCA, COAD, ESCA, OV, STAD, NSCLC, BRCA, LIHC, PAAD, and PRAD. We incorporated the above CTS-MS patterns into a logistic regression algorithm to propose a diagnosis model for each tumor type (Figure 2A). We further applied the classifier to predict the tissue of origin in an independent validation dataset with 2,580 additional samples (Supplementary Tables S1, S2). The classifier achieved an accurate classification decision, in which the area under the curve (AUC) ranged from 76 to 93% in different cancer types (Figures 2B,C). The AUC was relatively higher in cancer types with distinctive MS, such as BLCA (93%), COAD (92.5%), and ESAD (92.5%). However, PRAD was confused with other tumors, possibly due to the lack of specific MS patterns (Supplementary Figure S3A). Furthermore, we divided our validation dataset into three groups, including young, middle-aged, and elder samples. Results showed that the performance of our model remained stable across different age groups (Supplementary Figures S3B–D)*.* To evaluate the efficacy of MS in inferring primary tumor sites across different populations, we validated our model in an Asian population. We found that the AUC of our model in predicting the tumor origin of the Asian population was higher than 0.7, indicating that our model is stable in different populations (Figure 2D). Thus, the above results suggested that CTS-MS were robust candidate biomarkers for the differential diagnosis of various cancer types.

### Mutational Signature Patterns of Primary Cancers Maintain in Metastatic Sites

**FIGURE 3:**
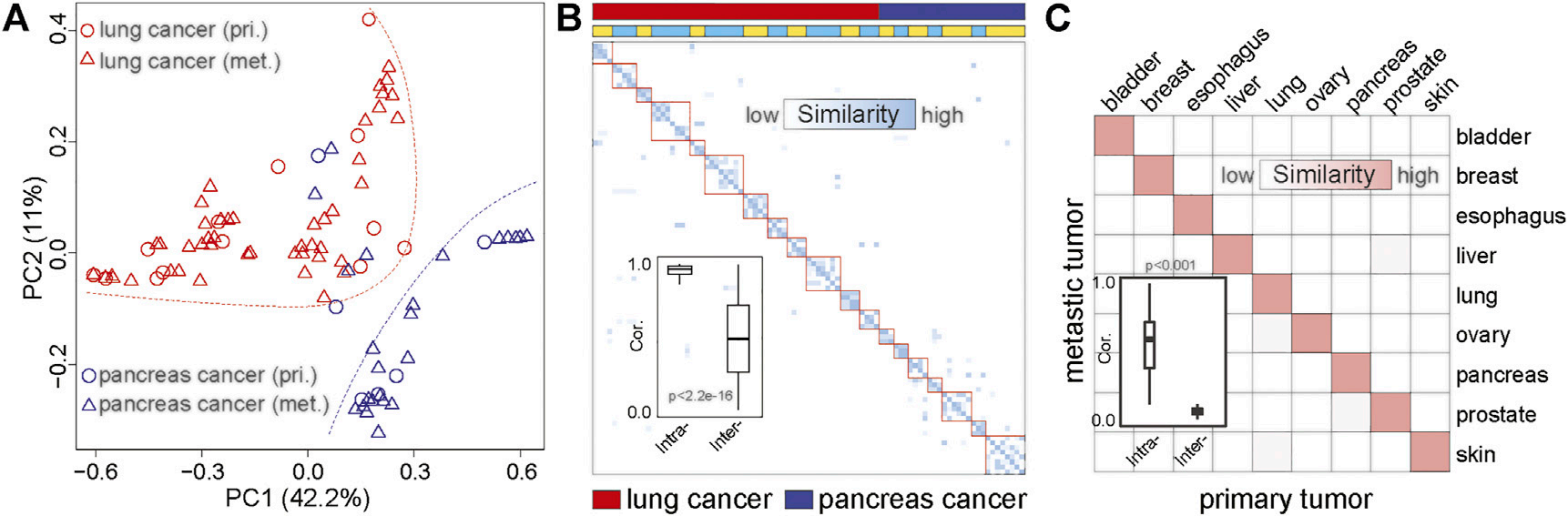
The similarity between metastatic and primary tumors based on MS. **(A)** PCA based on the MS of matched primary and metastatic cancers. The red dots represent the primary lung cancer, the red triangles represent metastatic lung cancer, the blue dots represent the primary pancreatic cancer, and the blue triangles represent metastatic pancreatic cancer. The red dotted line indicates the distribution area of lung cancer, and the blue dotted line indicates the distribution area of pancreatic cancer. pri., primary cancer; met., metastatic cancer. **(B)** The similarity between primary cancer and metastatic cancer based on MS. The darker the color, the higher the similarity. The first line indicates the tumor type. Red represents lung cancer, and blue represents pancreatic cancer. The second line shows the origin of the sample. The same color indicates that the sample is from the same patient. **(C)** The correlation between primary and metastatic cancer based on MS in common cancer. The darker the color, the higher the similarity. The boxplot shows the similarity between the primary and metastatic tumors of the same tumor type and the similarity between the primary and metastatic tumors among different tumor types.

Identification of the primary location of metastatic tumors is essential for precision treatment. To further evaluate the ability of MS to trace tumor location, we performed principal component analysis (PCA) on matched primary and metastatic cancers from 89 lesions (20 patients), including 30 pancreatic cancer and 59 lung cancers (Supplementary Table S3). We found that the samples were clustered by tumor origins (Figure 3A; Supplementary Figure S4). This result was consistent with the study from Connor et al., who found that the MS patterns between primary and metastatic tumors were similar (Connor et al., 2017). Furthermore, different tumor sites from the same individual also showed the same MS pattern (Figure 3B; Supplementary Figure S5). We compared the MS patterns in matched primary and metastatic cancers and observed high MS consistency between primary cancers and paired metastatic lesions (normalization score > 0.95, Figures 3A,B). However, the discrimination efficiency based on the original mutation spectrum was lower than that of MS, suggesting that MS can reveal the tissue origin of tumors more effectively than somatic mutations (Supplementary Figure S6).

To further validate the similarities between MS across the primary and metastatic tumors, we collected whole-exome data of primary and metastatic tumors of nine cancer types from the previous study (Zhao et al., 2016). We systematically analyzed the homogeneity between metastatic and primary cancer among nine cancer types. As shown in Figure 3C, high MS similarities were observed in the primary and metastatic tumor from the same tissue-of-origin (similarity > 0.9), which was significantly higher than the similarity among different cancer types (Wilcoxon rank-sum test *p* < 0.01). Therefore, our result revealed the high homogeneity of MS among the metastases and primary cancers from the same tissue, indicating that MS was a potential molecular marker for tracing the tissue of origin for metastatic cancers.

### Cancer-Type-Specific-Mutational Signatures can Help Identify the Tissue of Origin for Metastatic Cancers

**FIGURE 4:**
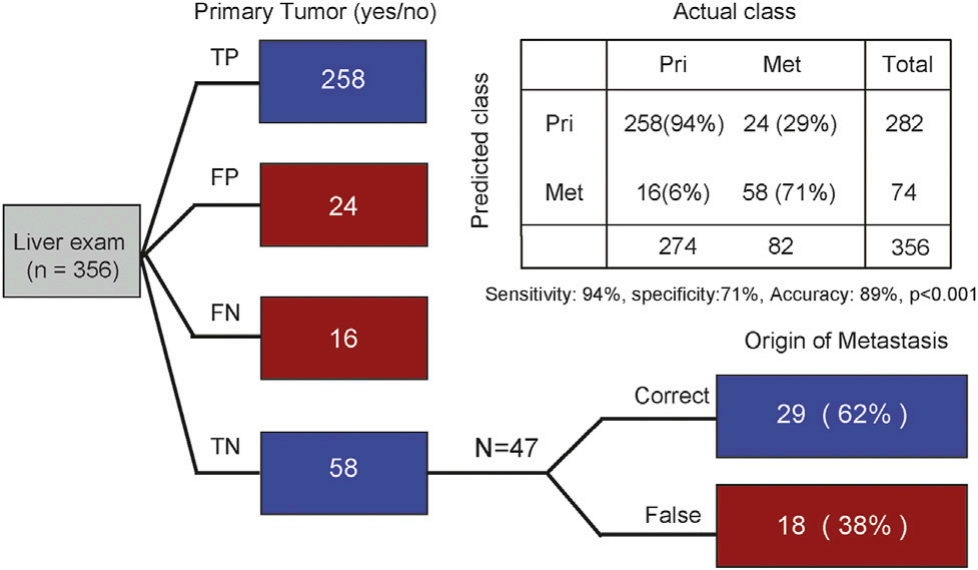
Tracing the origin of metastatic tumor based on MS. The first column distinguishes whether it is primary liver cancer. The second column traces the origin of metastatic cancer. TP, true positive; FP, false positive; FN, false negative; TN, true negative. Indicated are sample numbers and detection rates in percentages.

According to the above results, we next sought to evaluate whether CTS-MS was a stable and effective molecular marker for predicting the tissue origin of metastatic cancers. Liver is the most common site of distant metastasis in solid tumors (Riihimaki et al., 2016; Dasari et al., 2017). There is a pressing need for accurate tracing of original tissues (Varghese et al., 2017). We validated the ability of the CTS-MS to identify the tissue origin for metastatic tumor samples in an independent validation dataset that combined a series of 282 primary liver cancer with 74 liver metastatic tumors originating from other organs, including breast, prostate, and esophagus. Firstly, our model accurately distinguished the primary liver cancer and liver metastasis cancer originating from other organs (accuracy: 89%, sensitivity is 94%, specificity is 71%, Figure 4). Then, we determined the origin of cancer metastasized to the liver. We identified the origins of metastases with 62% accuracy, in which 75% of breast cancers were correctly classified. And we predicted esophageal cancer with 67% accuracy. However, we only predicted the origin of prostate cancer with 20% accuracy, probably due to the absence of PRAD-specific CTS-MS (Figure 1E; Supplementary Figure S3). Therefore, these results demonstrated that CTS-MS could help identify the tissue of origin for metastatic cancers.

### Cancer-Type-Specific-Mutational Signatures Analysis of Plasma cfDNA Enables Cancer Classification

**FIGURE 5:**
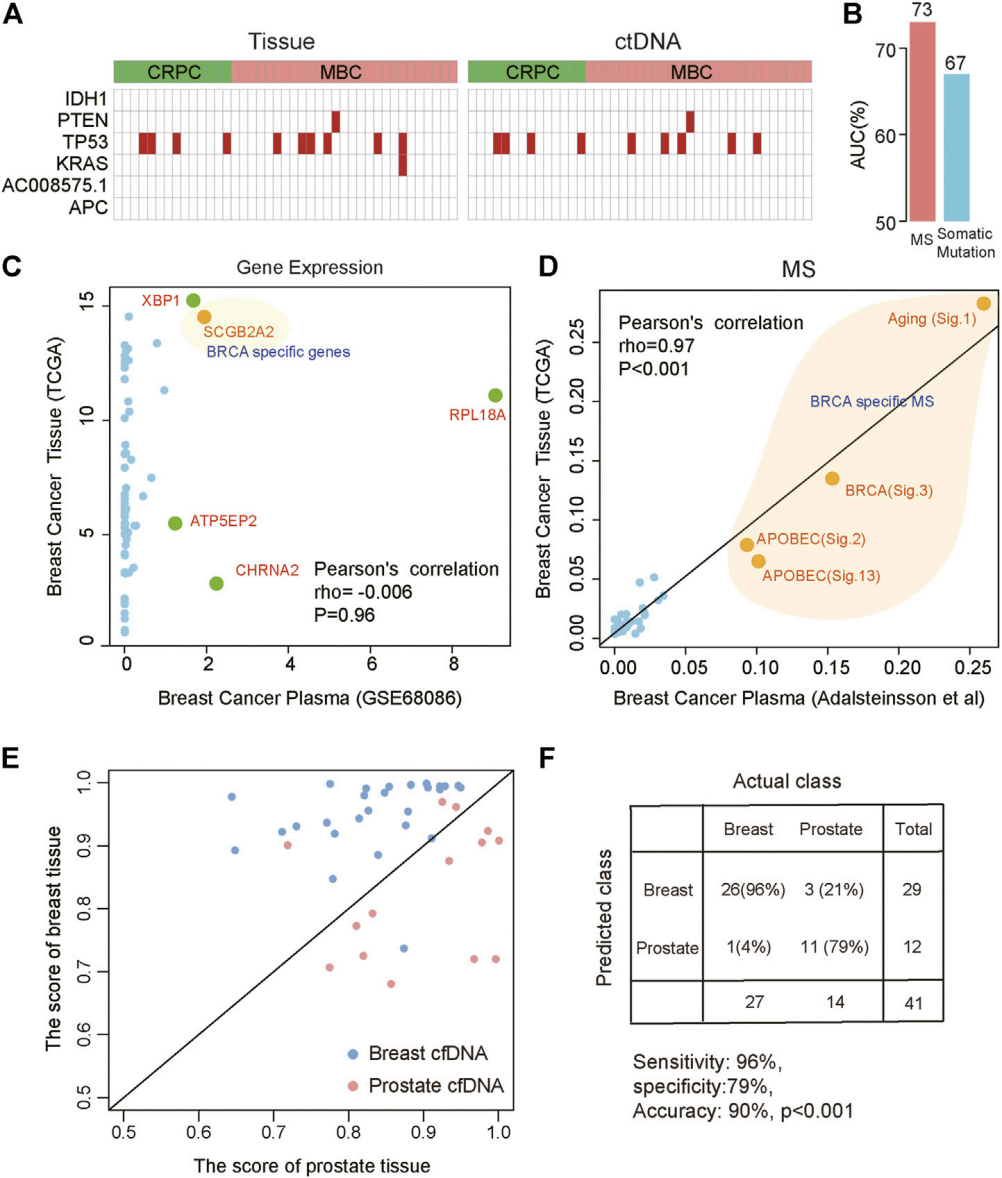
Distinguishing different cancer types based on the MS patterns and somatic mutations called from plasma ctDNA data. **(A)** The specific mutations in tissue (left) and ctDNA (right). Red indicates that mutation was detected. CRPC: prostate cancer; MBC: metastatic breast cancer. **(B)** The efficiency of MS and somatic mutations to distinguish lung cancer patients from benign lung nodules patients from ctDNA data. **(C,D)** The correlation between plasma and tissue in breast cancer based on gene expression **(C)** and MS **(D)**. Orange indicates breast cancer-specific markers. **(E,F)** Combined MS patterns and somatic mutations called from plasma ctDNA data distinguish breast cancer and prostate cancer. The red points represent prostate cancer and the blue points represent breast cancer. The horizontal axis represents the score of the prostate cancer model and the vertical axis represents the score of the breast cancer model.

The advent of non-invasive molecular profiling of plasma cell-free DNA (cfDNA) raises the possibility of inferring a suggested diagnosis in cancer screening. To assess the potential of MS for tracing the tumor origin based on plasma samples, we compared the MS patterns between cfDNA and matched breast and prostate tumor biopsies (Adalsteinsson et al., 2017). We found a high concordance of MS patterns between cfDNA and tissue (Spearman correlation, rho = 0.82, *p* < 0.001). Somatic mutation and gene expression have been used to predict cancer origins (He et al., 2020a; He et al., 2020b). To explore the efficiency of somatic mutation and gene expression in predicting the tumor origin from blood, we also compared the somatic mutation patterns and gene expression patterns between cfDNA and tumor tissue. We used the cancer-type specific genes (IDH1, PTEN, TP53, KRAS, AC008575.1, APC) in TOOme (He et al., 2020b) to evaluate the performance of somatic mutations detected in tissue or ctNDA for identifying the tumor tissue origin. We found that somatic mutations were detected in 26.8% (11/41) of tissue samples using these genes. The performance was even lower in paired ctDNA samples, with only 24.4% (10/41) detection rate (Figure 5A). Importantly, these gene mutations cannot distinguish breast cancer from prostate cancer based on these gene mutations. Thus, the above observations indicated that the performance of somatic mutations for inferring cancer tissue-of-origin was limited due to the substantial overlap in mutational profiles across different cancer types. Then, we compared the efficiency of MS and somatic mutations to identify the tumor from ctDNA, based on the somatic mutations detected from plasma of 111 lung cancer patients and 78 benign lung nodules patients (Chen et al., 2021). We found that MS was able to distinguish tumor from non-tumor patients better than mutations (AUC:0.73 vs. 0.67, Figure 5B). Next, we compared the expression similarity between tissues and plasma from breast cancer patients based on the genes used in TOOme. Our results indicated that the gene expression pattern differed between tissue and plasma of breast cancer. Almost all genes used to infer tumor tissue origin in TOOme were not expressed in plasma (Pearson correlation: −0.006, *p* = 0.96, Figure 5C). However, breast cancer-specific MS could be detected from ctDNA (Figure 5D). These analyses showed that MS is a reliable and stable biomarker for predicting the tumor tissue origin from plasma, compared with somatic mutation and RNA expression. Then, our model was further used to distinguish breast and prostate cancers based on MS patterns of cfDNA and achieved 71% accuracy. However, the model based on the mutation spectrum called cfDNA cannot distinguish these two tumor types (Supplementary Figure S7). We integrated the mutation profile of cfDNA and MS to build diagnosis models. The results showed that the performance of these diagnosis models had been significantly optimized. We predicted the tissue origin with 90% accuracy (sensitivity is 96%, specificity is 79%, Figures 5E,F). In summary, our analysis proved that the combination of MS and mutational profile was an available method to detect and localize cancers from peripheral blood.

## Discussion

Using the whole-exome sequencing data from tumors and cfDNA, we demonstrated that MS pattern was a potential approach for tumor detection and localization with high accuracy and robustness. First, we found that the somatic mutations in healthy individuals and inflammation patients were not associated with any known tumor initiation-related MS in the COSMIC database. This observation indicated that MS might separate healthy/inflammation patients and tumor patients. To further investigate whether MS could distinguish different tumor types, we analyzed the MS landscape of tumors from TCGA. Our results showed that different cancer types had specific MS patterns and validated this result in an independent dataset.

Moreover, using the CTS-MS, we could predict the tumor origin with high accuracy among primary and metastatic cancer. Notably, MS could better distinguish cancers from different tissues than somatic mutations. Finally, integrating the mutation profile and MS identified from cfDNA, we could predict the tissue origin of tumors with high accuracy. Therefore, our study showed that MS was a robust molecular marker for cancer diagnosis.

Lines of evidence indicate that the human body accumulates random mutations with age (Blokzijl et al., 2016; Hoang et al., 2016; Lodato et al., 2018; Zhang et al., 2019). The inflammation states accelerate this accumulation, such as ulcerative colitis, inflammatory bowel, or cirrhosis diseases (Brunner et al., 2019; Moore et al., 2020; Olafsson et al., 2020). The critical question is whether these accumulations of the somatic mutation have a functional impact or increased cancer risk. Our results indicated that the somatic mutations in healthy individuals had no functional relevance. In contrast, somatic mutations in tumor patients were functionally clustered and were related to specific biological processes, such as DNA damage repair deficiency. Our study showed that MS could distinguish between healthy individuals and tumor patients.

Some previous studies have reported that diverse ethnic populations have different mutational landscapes in the same type of cancer (Yao et al., 2016; Jia et al., 2017). However, the MS-based tumor tracing model in our study showed comparable performance between Asian and European and American populations for most of the tumor types, such as liver cancer, non-small cell lung cancer, and bladder cancer. This observation indicated that MS was a stable marker for predicting the tumor tissue origin in different populations. Consistently, Zhang et al. reported that MS patterns were shared in different populations with liver cancer, including Signature.5, Signature.22, and Signature.24 (Zhang et al., 2017; Zhang and Guan, 2021).

Notably, with one or more confirmed metastatic malignant lesions but the undetectable primary origin, cancers of unknown primary (CUP) make up 3–5% of total cancer diagnoses and have a very poor prognosis with a median survival of 6–16 months (Varadhachary and Raber, 2014; Conway et al., 2019). Refining the diagnostic classification of CUP patients can facilitate the selection of potentially effective therapies (Varghese et al., 2017). We found that the MS of the primary and metastatic cancers from identical tissue were highly consistent in whole-exome sequencing, indicating the tumor traceability of MS for metastatic cancers. We distinguished the malignant liver lesions originating from other tissues and primary liver tumors with high accuracy, indicating that our MS-based model could trace the origin of the metastatic tumor. Besides, MS inferred from cfDNA was highly compatible with tumor biopsies. Since liquid biopsy is increasingly used for cancer screening and diagnosis, our method may help infer the tissue origin by cfDNA detection.

In this study, although we demonstrated the potential diagnostic value of MS in determining the cancer origin by two independent datasets, more samples needed to be included to train more robust and precise models. Besides, only a limited number of MS have been discovered in the human tissue. The etiology and exposure factors of the majority of MS remain unclear currently (Alexandrov et al., 2013). With the development of sequencing technology, more reliable cancer-related MS will be determined, allowing more features could be included in our model to achieve higher accuracy.

In conclusion, we showed that MS was a reliable biomarker for tumor detection and localization. Our study will provide vital information for clinical diagnosis and tracing tumor origin for cancers without known primary sites.

## Data Availability

The original contributions presented in the study are included in the article/Supplementary Material, further inquiries can be directed to the corresponding authors.
