# Supplementary figures and images for: Detection and Localization of Solid Tumors Utilizing the Cancer-Type-Specific Mutational Signatures
Source: Front Bioeng Biotechnol. 2022 Apr 25;10:883791. doi: 10.3389/fbioe.2022.883791 (PMC9081532; doi:10.3389/fbioe.2022.883791)

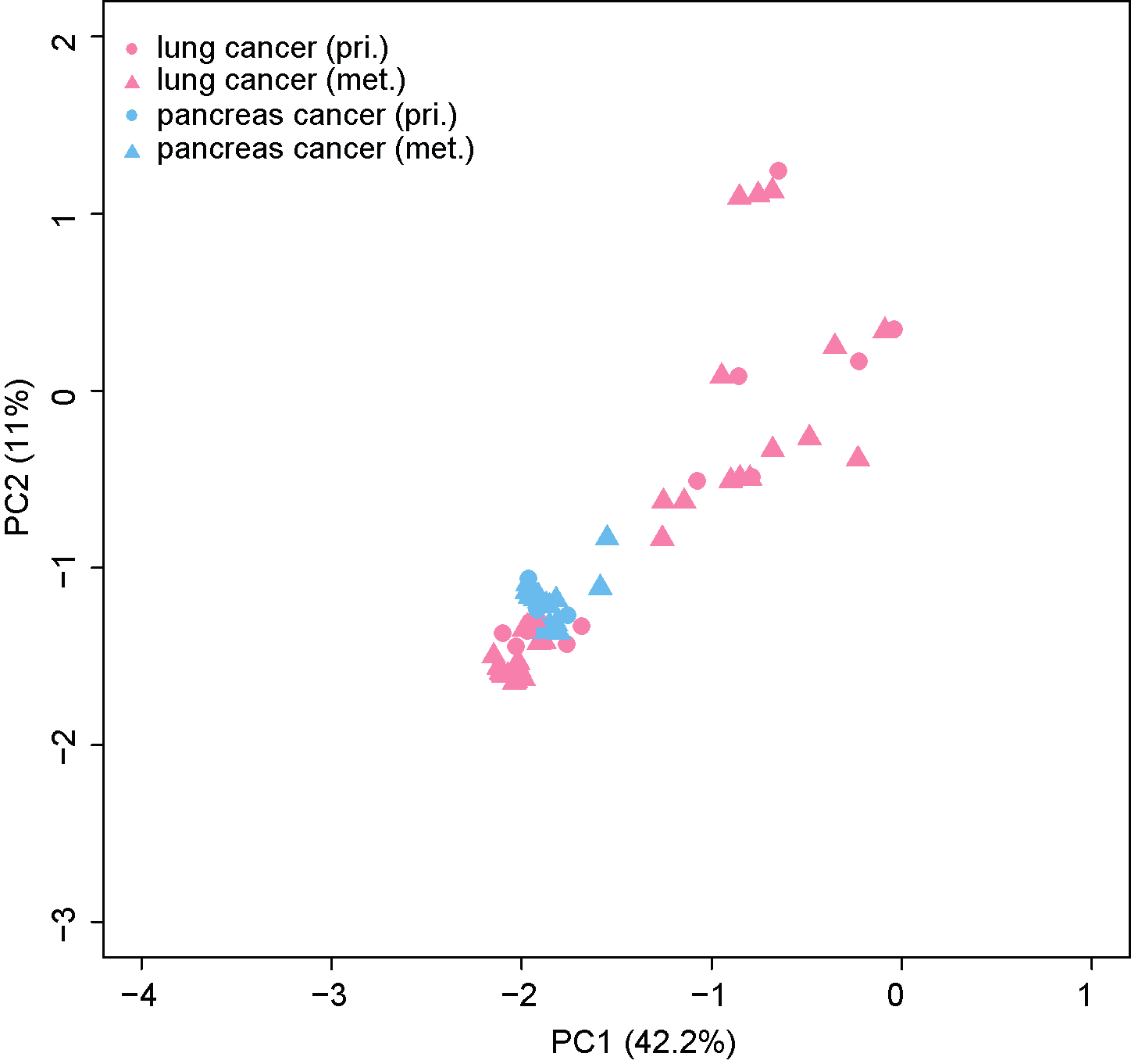

Supplement: Supplementary file 1 [file Image6.TIF]

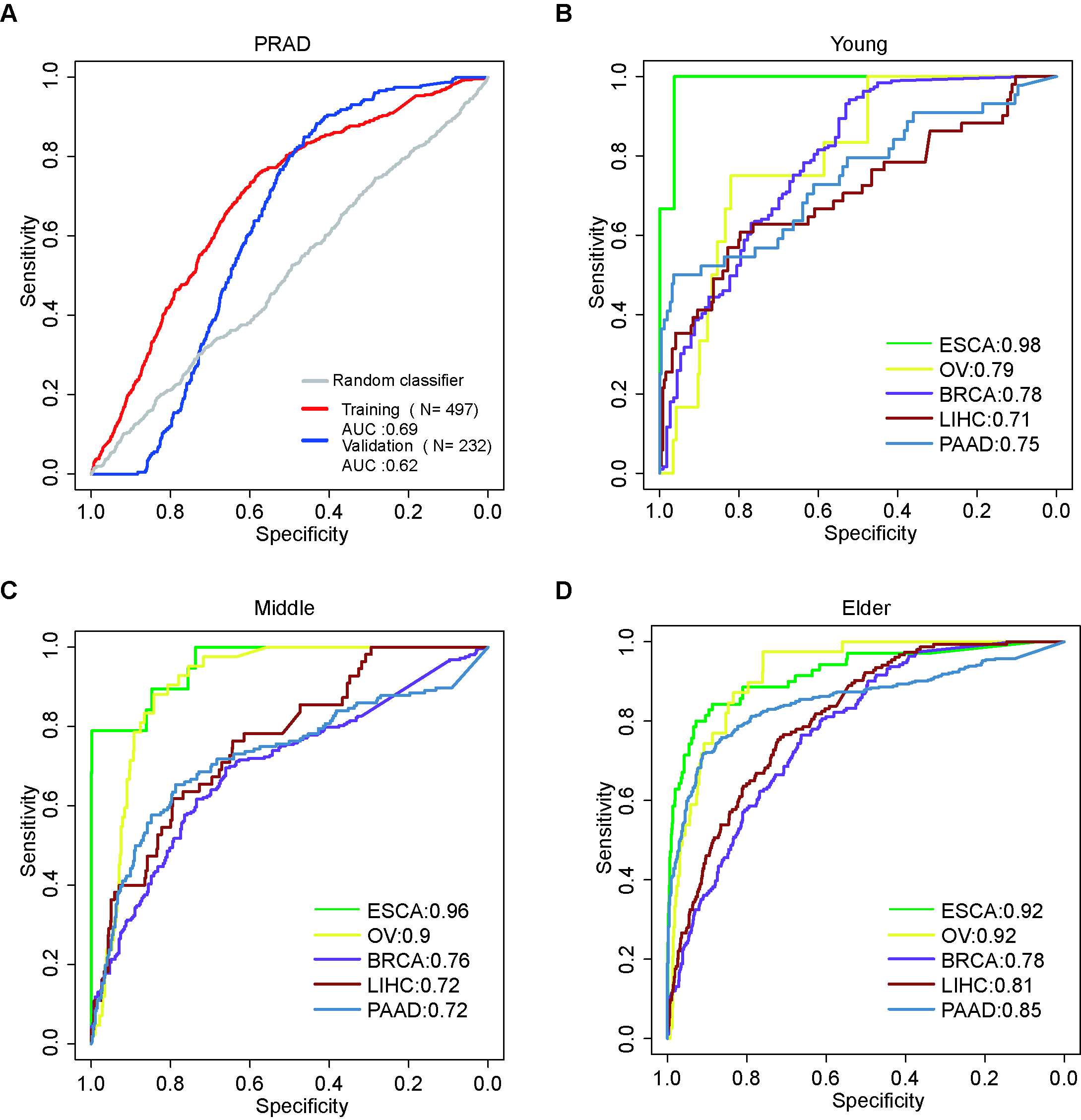

Supplement: Supplementary file 3 [file Image3.TIF]

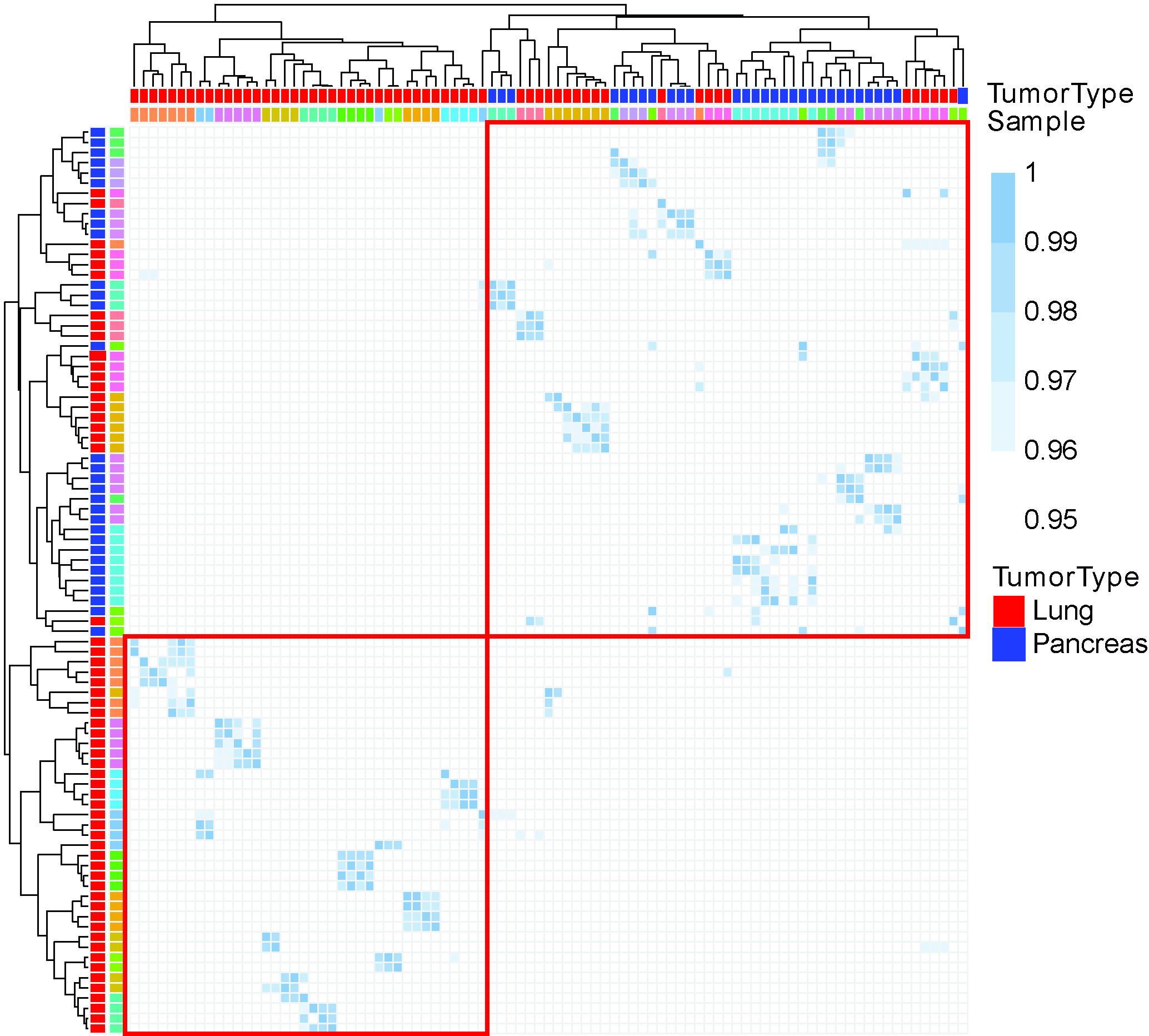

Supplement: Supplementary file 4 [file Image4.TIF]

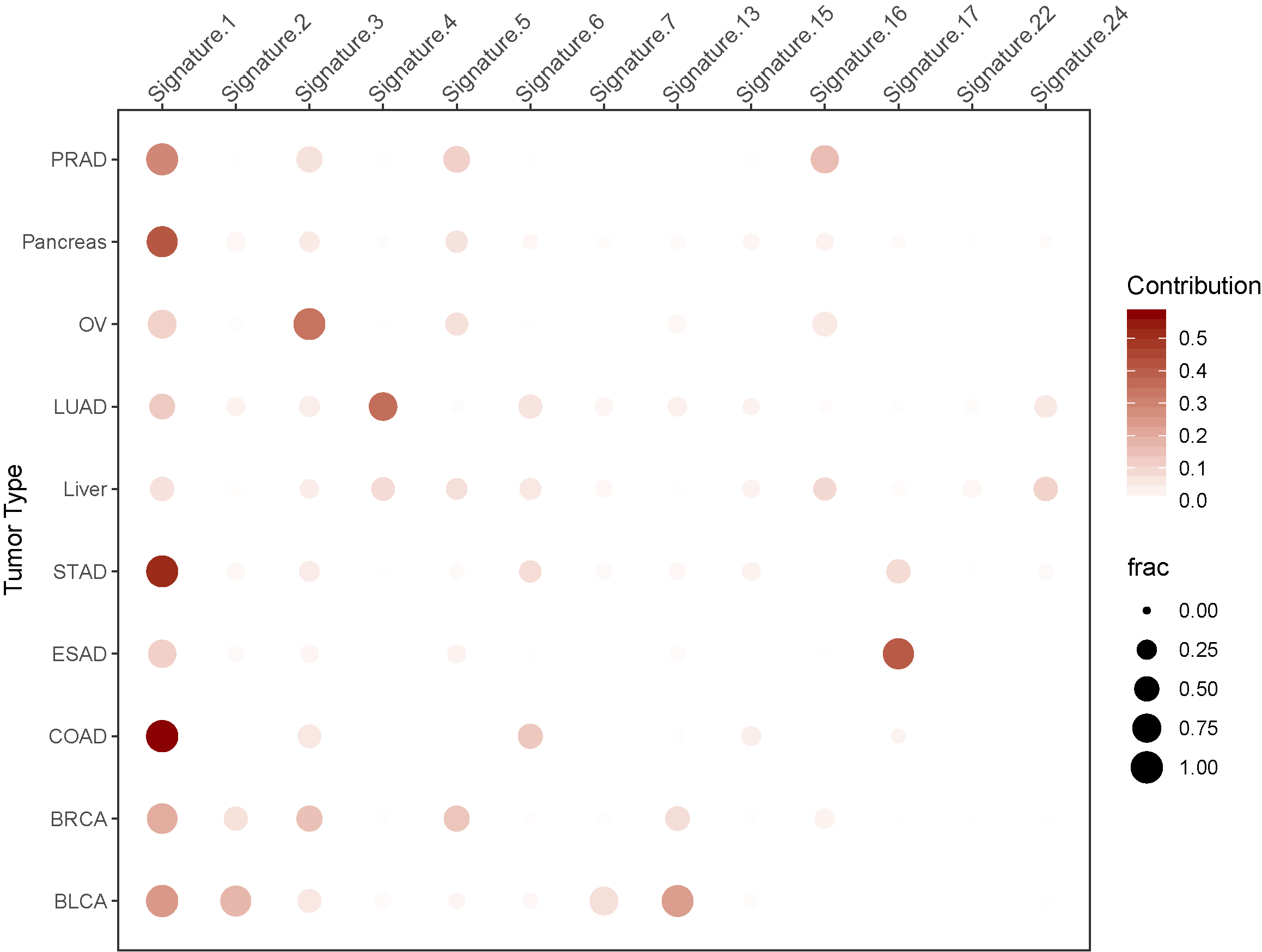

Supplement: Supplementary file 5 [file Image2.TIF]

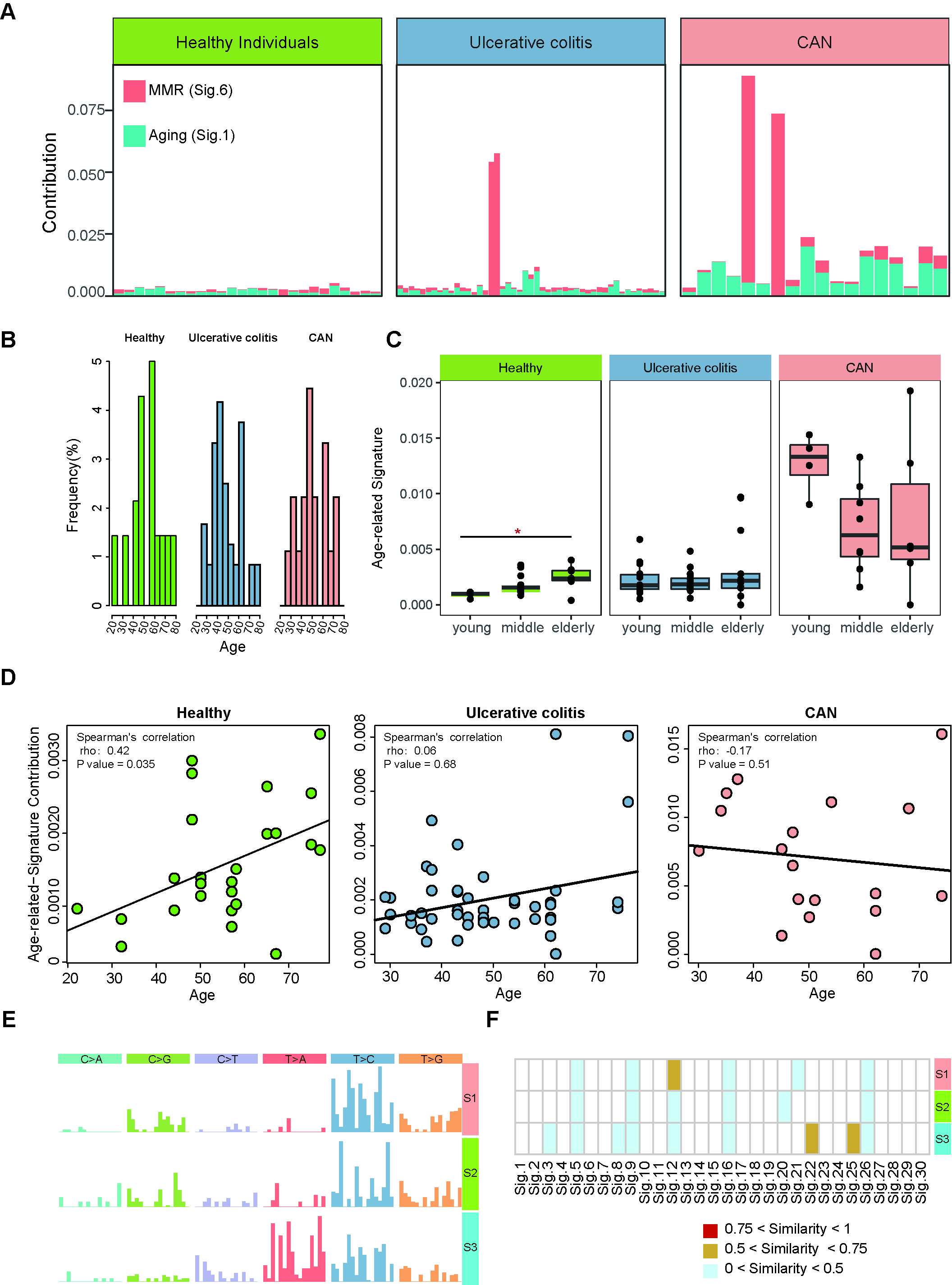

Supplement: Supplementary file 6 [file Image1.TIF]

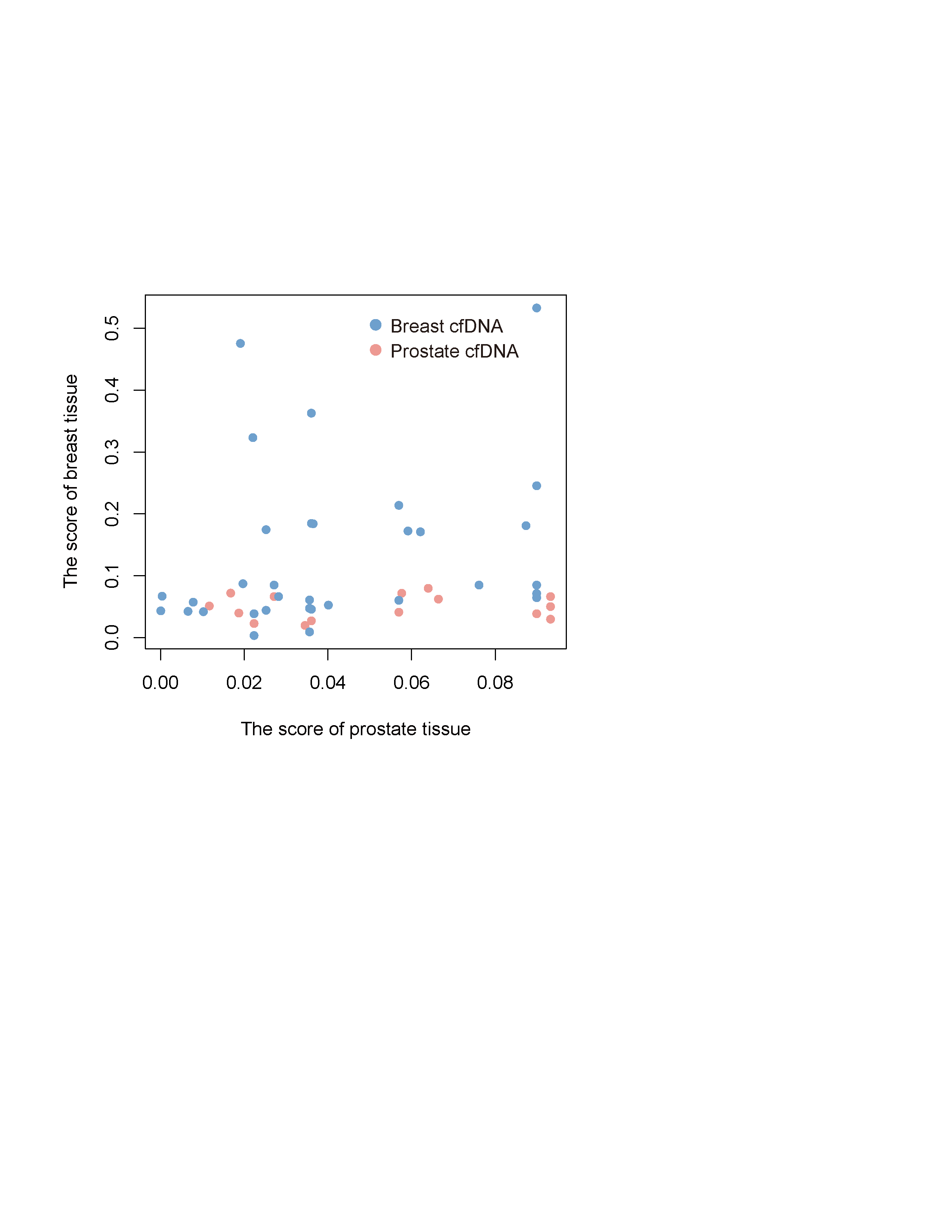

Supplement: Supplementary file 7 [file Image7.TIF]

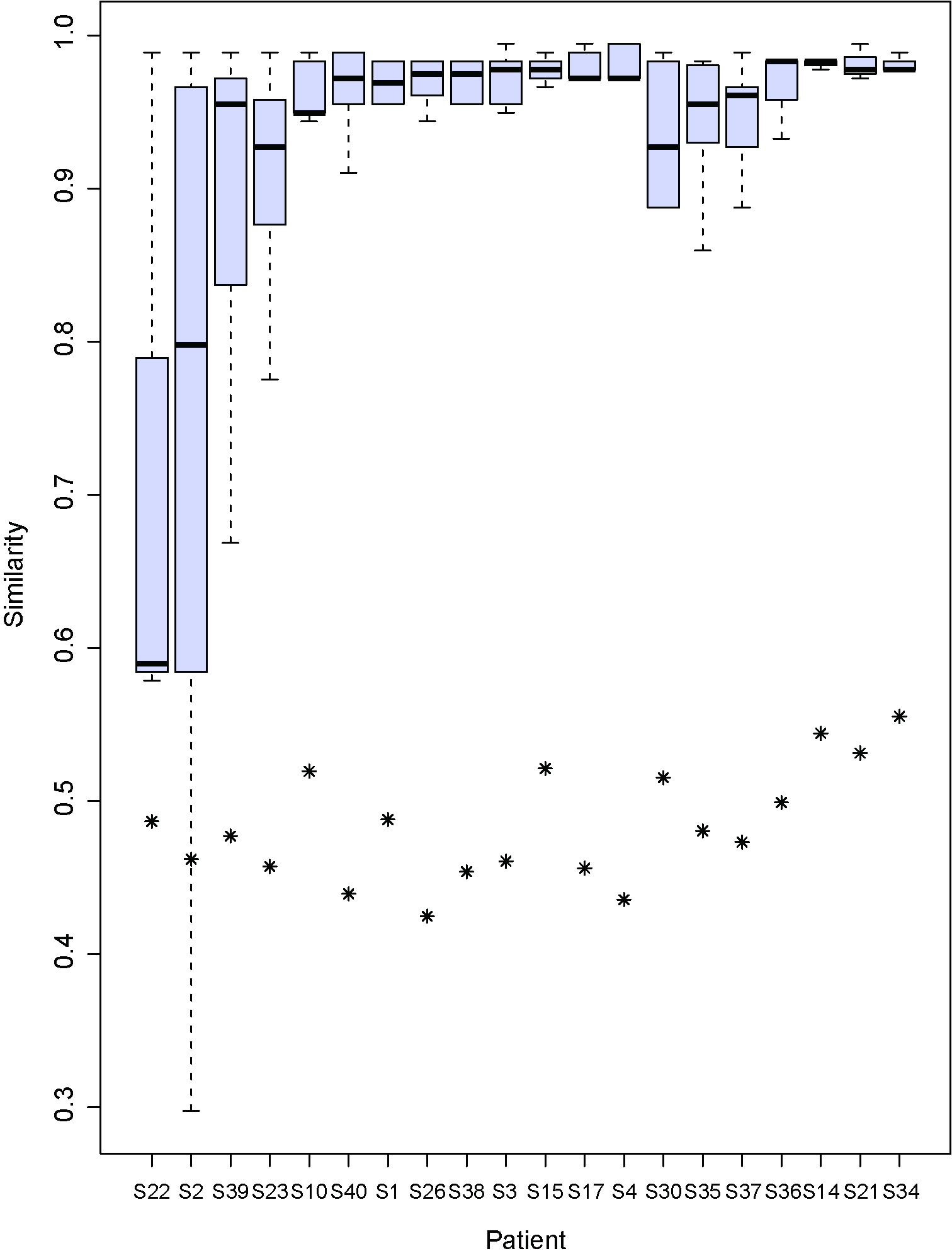

Supplement: Supplementary file 10 [file Image5.TIF]
