# Supplementary material for: Detection and Localization of Solid Tumors Utilizing the Cancer-Type-Specific Mutational Signatures
Source: Front Bioeng Biotechnol. 2022 Apr 25;10:883791. doi: 10.3389/fbioe.2022.883791 (PMC9081532; doi:10.3389/fbioe.2022.883791)
Supplement: Supplementary file 2 [file Table1.DOCX]

**Supplementary table 1: The number of samples and the data source of each cancer type**

| Anatomical site | Number (Total^#^) | Source |
| --- | --- | --- |
| Bladder | 558 | TCGA/ICGC |
| Colorectal | 597 | TCGA |
| Esophagus | 632 | TCGA/ICGC |
| Ovary | 540 | TCGA/ICGC |
| Stomach | 516 | TCGA/ICGC |
| Lung | 1391 | TCGA/ICGC |
| Breast | 1893 | TCGA/ICGC |
| Liver | 813 | TCGA/ICGC |
| Pancreas | 959 | TCGA/ICGC |
| Prostate | 849 | TCGA/ICGC |
| Skin | 478 | TCGA |

^#^The sum of samples included in this study for each cancer, including the whole-exome data of primary tumors, metastases, and plasma cfDNA.
