# Supplementary material for: Detection and Localization of Solid Tumors Utilizing the Cancer-Type-Specific Mutational Signatures
Source: Front Bioeng Biotechnol. 2022 Apr 25;10:883791. doi: 10.3389/fbioe.2022.883791 (PMC9081532; doi:10.3389/fbioe.2022.883791)
Supplement: Supplementary file 8 [file Table2.DOCX]

**Supplementary table 2: The specificity and sensitivity of the training and validation dataset for each primary cancer model.**

| Anatomical site | Training cohort (N=5001) | Validation cohort (N=2580) | Sensitivity | Specificity |
| --- | --- | --- | --- | --- |
| Bladder | 412 | 30 | 0.97 | 0.91 |
| Colorectal | 398 | 12 | 1 | 0.78 |
| Esophagus | 184 | 203 | 0.83 | 0.88 |
| Ovary | 435 | 93 | 0.88 | 0.79 |
| Stomach | 439 | 77 | 0.81 | 0.82 |
| Lung | 1108 | 182 | 0.71 | 0.94 |
| Breast | 985 | 741 | 0.69 | 0.75 |
| Liver | 364 | 274 | 0.65 | 0.78 |
| Pancreas | 179 | 736 | 0.64 | 0.82 |
| Prostate | 497 | 232 | 0.88 | 0.43 |
