# Supplementary material for: Detection and Localization of Solid Tumors Utilizing the Cancer-Type-Specific Mutational Signatures
Source: Front Bioeng Biotechnol. 2022 Apr 25;10:883791. doi: 10.3389/fbioe.2022.883791 (PMC9081532; doi:10.3389/fbioe.2022.883791)
Supplement: Supplementary file 9 [file Table3.DOCX]

**Supplementary table 3: The number of primary and metastases samples in each cancer type**

| Anatomical site | Primary  (N=4551) | Metastases (N=359) | | Primary-Metastatic Paired | Source  (PMID) |
| --- | --- | --- | --- | --- | --- |
| Bladder | 412 | | 13 | No | 28783718;29259186 |
| Breast | 985 | | 92 | No | 28783718 |
| Esophagus | 184 | | 17 | No | 28783718 |
| Liver | 364 | | 12 | No | 28783718 |
| Ovary | 435 | | 12 | No | 28783718 |
| Prostate | 497 | | 92 | No | 28783718 |
| Skin | 468 | | 10 | No | 28783718 |
| Lung | 1008 | | 27 | No | 28783718 |
| Lung | 13 | | 46 | YES | 26858460 |
| Pancreas | 179 | | 14 | No | 28783718 |
| Pancreas | 6 | | 24 | YES | 26858460 |
